# Supplementary figures and images for: Climacteric women’s perspectives on menopause and hormone therapy: Knowledge gaps, fears, and the role of healthcare advice
Source: PLoS One. 2025 May 9;20(5):e0316873. doi: 10.1371/journal.pone.0316873 (PMC12063881; doi:10.1371/journal.pone.0316873)

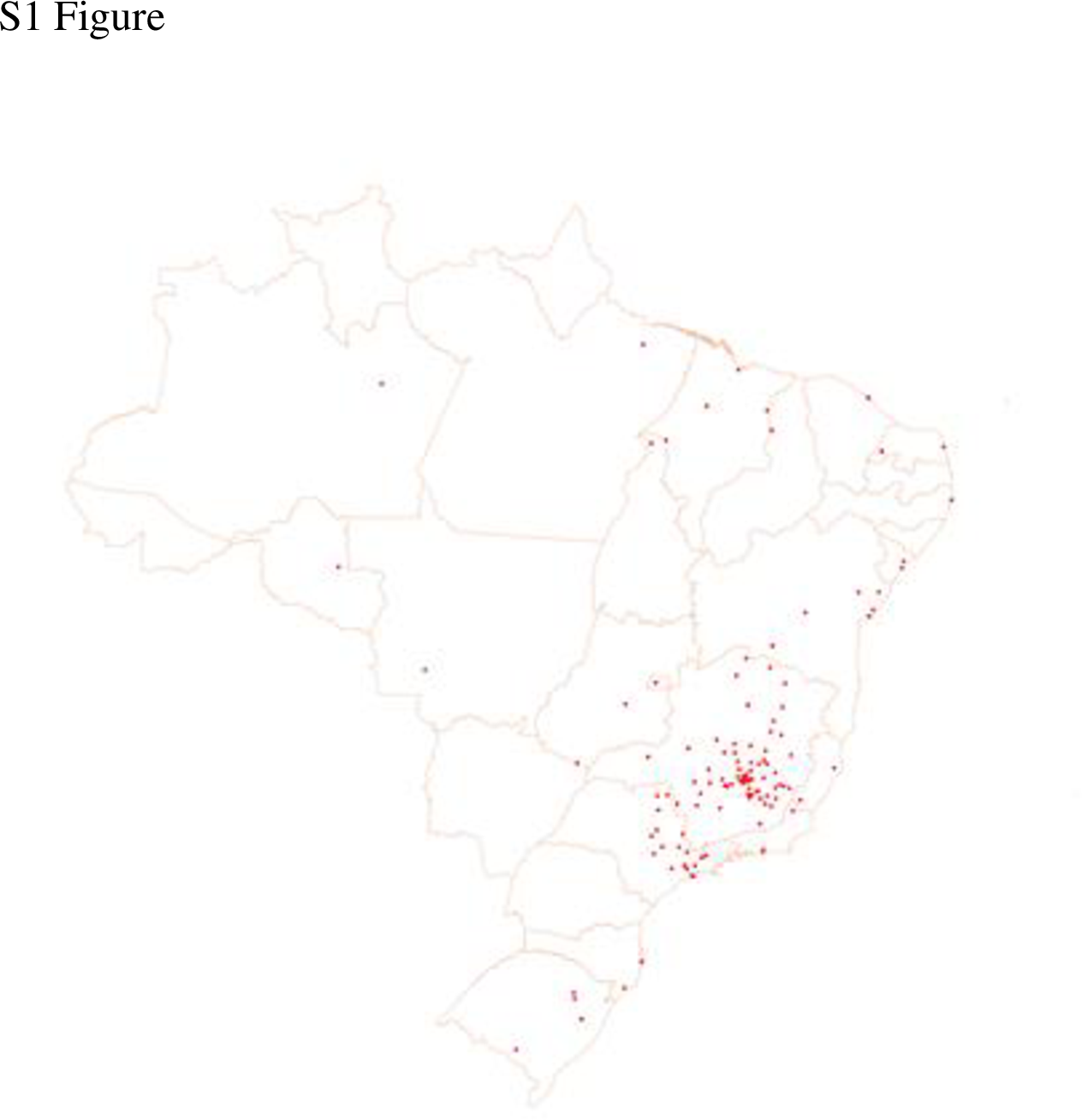

Supplement: S1 Fig — (TIF) [file pone.0316873.s001.tif]

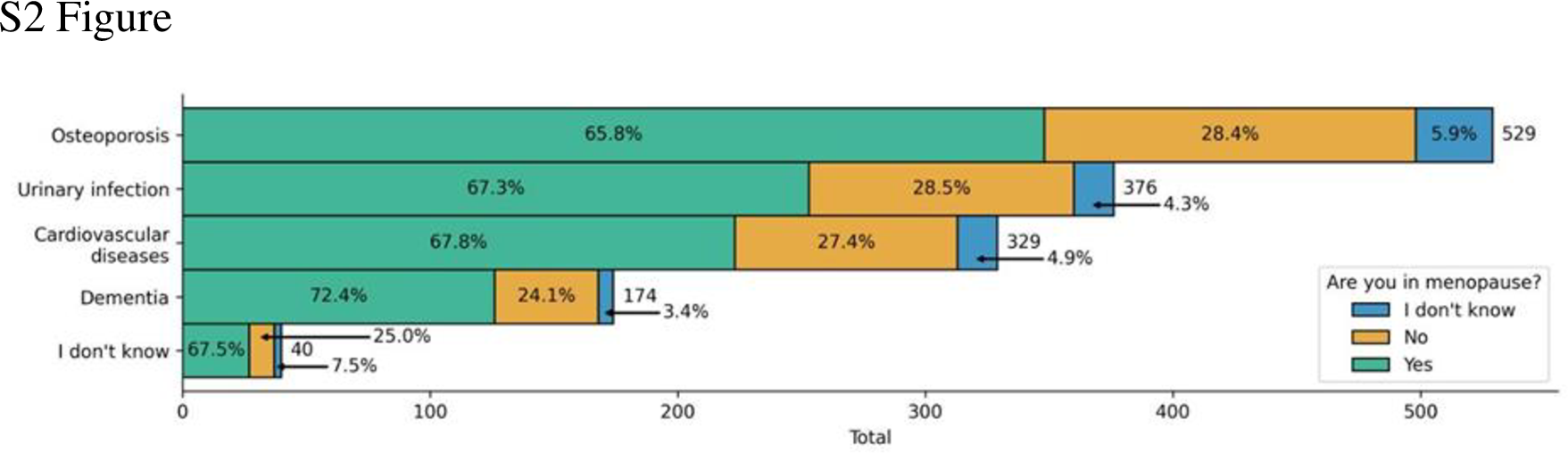

Supplement: S2 Fig — (TIF) [file pone.0316873.s002.tif]
