# Supplementary material for: Climacteric women’s perspectives on menopause and hormone therapy: Knowledge gaps, fears, and the role of healthcare advice
Source: PLoS One. 2025 May 9;20(5):e0316873. doi: 10.1371/journal.pone.0316873 (PMC12063881; doi:10.1371/journal.pone.0316873)
Supplement: S1 Table — (DOCX) [file pone.0316873.s003.docx]

**S1Table.** Knowledge of menopause, according to menopause condition.

| **Characteristics** | **Total**  **(n=1139)** | **Post-menopausal women**  **(n=631, 55.40%)** | **Pre-menopausal**  **women**  **(n=508, 44.60%)** | *p - value* |
| --- | --- | --- | --- | --- |
| **Have you ever heard about menopause?, n (%)** | 1133 (99.47) | 629 (99.68) | 504 (99.21) | 0.276^#^ |
| **Do you know about menopause symptoms?, n (%)** | 1068 (94.26) | 618 (98.25) | 450 (89.29) | <0.001^#^ |
| **Conditional answer for yes (n=1068)** | **(n=1068)** | **618 (57.87%)** | **450 (42.13%)** |  |
| Hot flashes, n (%) | 1038 (97.19) | 598 (96.76) | 440 (97.78) | <0.001^#^ |
| Vaginal dryness, n (%) | 719 (67.32) | 464 (75.08) | 255 (56.67) | <0.001^#^ |
| Mood swings, n (%) | 723 (67.70) | 435 (70.39) | 288 (64.00) | <0.001^#^ |
| Sleeplessness, n (%) | 749 (70.13) | 468 (75.73) | 281 (62.44) | <0.001^#^ |
| Menstrual irregularities, n (%) | 643 (60.21) | 365 (59.06) | 278 (61.78) | <0.001* |
| Loss of libido, n (%) | 621 (58.15) | 386 (62.46) | 235 (52.22) | 0.001^#^ |
| **How did you find out about menopause symptoms?, n (%)** | (n=1133) |  |  |  |
| Physician, n (%) | 819 (72.29) | 518 (82.35) | 301 (59.72) | <0.001^#^ |
| Relatives or friends, n (%) | 835 (73.70) | 444 (70.59) | 391 (77.58) | <0.001^#^ |
| Internet, n (%) | 486 (42.89) | 255 (40.54) | 231 (45.83) | <0.001^#^ |
| Television, n (%) | 331 (29.21) | 186 (29.57) | 145 (28.77) | <0.001^#^ |
| Magazines, n (%) | 218 (19.24) | 134 (21.30) | 84 (16.67) | <0.001^#^ |
| Newspaper, n (%) | 194 (17.12) | 111 (17.65) | 83 (16.47) | <0.001^#^ |
| Teachers, n (%) | 119 (10.50) | 62 (9.86) | 57 (11.31) | <0.001^#^ |
| Nurse, n (%) | 127 (11.21) | 63 (10.02) | 64 (12.70) | <0.001^#^ |
| Religious leaders, n (%) | 39 (3.44) | 29 (4.61) | 10 (1.98) | <0.001^#^ |
| I do not remeber, n (%) | 12 (1.06) | 7 (1.11) | 5 (0.99) | <0.001^#^ |
| **How satisfied were you with the information on menopause that your physician had given? n=1133** |  |  |  |  |
| Completely or reasonably satisfied, n (%) | 343 (30.27) | 226 (35.93) | 117 (23.21) | <0.001^#^ |
| I am reasonably satisfied, n (%) | 305 (26.92) | 180 (28.62) | 125 (24.80) |  |
| Neither dissatisfied nor satisfied, n (%) | 263 (23.21) | 107 (17.01) | 156 (30.95) |  |
| I'm a little dissatisfied, n (%) | 111 ( 9.80) | 55 ( 8.74) | 56 (11.11) |  |
| I am totally dissatisfied, n (%) | 111 ( 9.80) | 61 ( 9.70) | 50 ( 9.92) |  |
| Any knowledge regarding illness related to menopause, n (%) | 997 (87.53) | 580 (91.92) | 417 (82.09) | <0.001^#^ |
| **Conditional answer for yes (n= 654)** | 654 | **432 (66.06%)** | **222 (33.94%)** |  |
| **Risk of having an illness related to menopause, n (%)** | 654 (57.72) | 432 (68.68) | 222 (44.05) | <0.001^#^ |
| Cardiovascular diseases, n (%) | 329 (50.31) | 223 (51.62) | 106 (47.75) | <0.001^#^ |
| Osteoporosis, n (%) | 529 (80.89) | 348 (80.56) | 181 (81.53) | <0.001^#^ |
| Dementia, n (%) | 174 (26.61) | 126 (29.17) | 48 (21.62) | <0.001^#^ |
| Urinary infection, n (%) | 376 (57.49) | 253 (58.56) | 123 (55.41) | <0.001^#^ |
| I don't know, n (%) | 40 (6.12) | 27 (6.25) | 13 (5.86) | <0.001^#^ |
| **How concerned about the risk of osteoporosis (n=653)** |  |  |  | p=0.07^##^ |
| I don´t worry, n (%) | 29 (4.44) | 24 (5.56) | 5 (2.26) |  |
| I'm worry a little, n (%) | 130 (19.91) | 75 (17.36) | 55 (24.89) |  |
| I'm not worried, n (%) | 17 (2.60) | 8 (1.85) | 9(4.07) |  |
| I'm worry a lot, n (%) | 238 (36.45) | 174 (40.28) | 64 (28.96) |  |
| I'm extremely worried, n (%) | 239 (36.60) | 151 (34.95) | 88 (39.82) |  |
| **How concerned about the risk of cardiovascular disease (n=654)** |  |  |  | p=0.76^##^ |
| I don´t worry, n (%) | 43 (6.57) | 31 (7.18) | 12 (5.41) |  |
| I'm worry a little, n (%) | 112 (17.13) | 72 (16.67) | 40 (18.02) |  |
| I'm not worried, n (%) | 17 (2.60) | 9 (2.08) | 8 (3.60) |  |
| I'm worry a lot, n (%) | 232 (35.47) | 162 (37.50) | 70 (31.53) |  |
| I'm extremely worried, n (%) | 250 (38.23) | 158 (36.57) | 92 (41.44) |  |
| **How concerned about the risk of vaginal dryness (n=653)** |  |  |  | p=0.37^##^ |
| I don't worry, n (%) | 52 (7.96) | 41 (9.49) | 11 (4.98) |  |
| I'm worry a little, n (%) | 134 (20.52) | 85 (19.68) | 49 (22.17) |  |
| I'm not worried, n (%) | 33 (5.05) | 24 (5.56) | 9 (4.07) |  |
| I'm worry a lot, n (%) | 204 (31.24) | 141 (32.64) | 63 (28.51) |  |
| I'm extremely worried, n (%) | 230 (35.22) | 141 (32.64) | 89 (40.27) |  |
| **How concerned about the risk of loss of libido (n=653)** |  |  |  | p=0.19^##^ |
| I donVt worry, n (%) | 59 (9.04) | 50 (11.57) | 9 (4.07) |  |
| I'm worry a little, n (%) | 127 (19.45) | 78 (18.06) | 49 (22.17) |  |
| I'm not worried, n (%) | 34 (5.21) | 25 (5.79) | 9 (4.07) |  |
| I'm worry a lot, n (%) | 193 (29.56) | 129 (29.86) | 64 (28.96) |  |
| I'm extremely worried, n (%) | 240 (36.75) | 150 (34.72) | 90 (40.72) |  |
| **How concerned about the risk of mood swings (n=654)** |  |  |  | p=0.04^##^ |
| I don't worry, n (%) | 24 (3.67) | 21 (4.86) | 3 (1.35) |  |
| I'm worry a little, n (%) | 86 (13.15) | 63 (14.58) | 23 (10.36) |  |
| I'm not worried, n (%) | 19 (2.91) | 11 (2.55) | 8 (3.60) |  |
| I'm worry a lot, n (%) | 246 (37.61) | 165 (38.19) | 81 (36.49) |  |
| I'm extremely worried, n (%) | 279 (42.66) | 172 (39.81) | 107 (48.20) |  |
| **How concerned about the risk of breast cancer (n=653)** |  |  |  | p=0.51^##^ |
| I don't worry, n (%) | 21 (3.22) | 13 (3.01) | 8 (3.62) |  |
| I'm worry a little, n (%) | 69 (10.57) | 51 (11.81) | 18 (8.14) |  |
| I'm not worried, n (%) | 10 (1.53) | 5 (1.16) | 5 (2.26) |  |
| I'm worry a lot, n (%) | 237 (36.29) | 160 (37.04) | 77 (34.84) |  |
| I'm extremely worried, n (%) | 316 (48.39) | 203 (46.99) | 113 (51.13) |  |
| **Which of the following do you think women’s bodies naturally produce? (n=1138)** |  |  |  |  |
| Testosterone, n (%) | 188 (16.51) | 106 (16.80) | 82 (16.14) | p=0.767^#^ |
| Estrogens, n (%) | 686 (60.23) | 388 (61.49) | 298 (58.66) | p=0.333^#^ |
| Progesterone, n (%) | 641 (56.28) | 363 (57.53) | 278 (54.72) | p=0.343^#^ |
| I don´t know, n (%) | 300 (26.34) | 146 (23.14) | 154 (30.31) | p=0.006^#^ |
| **To the best of your knowledge, what do you think that happens with hormones after menopause?** |  |  |  | p<0.001^#^ |
| Decrease after menopause, n (%) | 940 (82.53) | 541 (85.74) | 399 (78.54) |  |
| Increase after menopause, n (%) | 57 (5.00) | 32 (5.07) | 25 (4.92) |  |
| Doesn’t affect production after menopause, n (%) | 6 (0.53) | 3 (0.48) | 3 (0.59) |  |
| I don´t know, n (%) | 136 (11.94) | 55 (8.72) | 81 (15.94) |  |
| **Do you know if there is a treatment for menopause symptoms?, n (%)** | 997 (87.53) | 580 (91.92) | 417 (82.09) | p<0.001^#^ |
| **What treatment(s) for menopause symptoms do you know?**  **Conditional answer for yes (n=997)** |  |  |  |  |
| Physical activities, n (%) | 686 (68.81) | 427 (73.62) | 259 (62.11) | p<0.001^#^ |
| Yoga, n (%) | 296 (29.69) | 190 (32.76) | 106 (25.42) | p<0.001^#^ |
| Acupuncture, n (%) | 226 (22.67) | 131 (22.59) | 95 (22.78) | p<0.001^#^ |
| Herbal treatments, n (%) | 450 (45.14) | 278 (47.93) | 172 (41.25) | p<0.001^#^ |
| Vitamin complexes, n (%) | 365 (36.61) | 214 (36.90) | 151 (36.21) | p<0.001^#^ |
| Hormone therapy, n (%) | 880 (88.26) | 511 (88.10) | 369 (88.49) | p<0.001^#^ |
| No treatment, n (%) | 10 (1.00) | 5 (0.86) | 5 (1.20) | p<0.001^#^ |
| I don´t know, n (%) | 27 (2.71) | 11 (1.90) | 16 (3.84) | p<0.001^#^ |
| **What do you know about Menopause Hormone Therapy (MHT)? (n=1138)** |  |  |  | p<0.001^#^ |
| I know a lot, n (%) | 80 (7.03) | 57 (9.05) | 23 (4.53) |  |
| I know a little, n (%) | 758 (66.61) | 439 (69.68) | 319 (62.80) |  |
| I don't know anything, n (%) | 300 (26.36) | 134 (21.27) | 166 (32.68) |  |
| **Do you know if there are negative effects of using hormonal therapy during menopause? (n=1139)** |  |  |  | p<0.001^#^ |
| Yes, n (%) | 715 (62.77) | 452 (71.63) | 263 (51.77) |  |
| No, n (%) | 113 (9.92) | 55 (8.72) | 58 (11.42) |  |
| I don't know, n (%) | 311 (27.30) | 124 (19.65) | 187 (36.81) |  |
| **Do you know if hormonal therapy to treat menopause is offered by the Brazilian health system (SUS)?** |  |  |  | p<0.001**^#^** |
| Yes, n (%) | 252 (22.12) | 163 (25.83) | 89 (17.52) |  |
| No, n (%) | 367 (32.22) | 219 (34.71) | 148 (29.13) |  |
| I don't know, n (%) | 520 (45.65) | 249 (39.46) | 271 (53.35) |  |
| **Do you know if and where the Brazilian Health System offers hormonal therapy to treat menopause?** |  |  |  | p=0.014 |
| It is offered at a discount by private health secure, n (%) | 18 (1.58) | 10 (1.58) | 8 (1.57) |  |
| Not offered anywhere n (%) | 32 (2.81) | 18 (2.85) | 14 (2.76) |  |
| I don't know, n (%) | 769 (67.52) | 405 (64.18) | 364 (71.65) |  |
| In the private system, n (%) | 21 (1.84) | 14 (2.22) | 7 (1.38) |  |
| In the public system (SUS), n (%) | 283 (24.85) | 173 (27.42) | 110 (21.65) |  |
| In public and private systems n (%) | 16 (1.40) | 11 (1.74) | 5 (0.98) |  |

*Kruskal-Wallis Test. ^#^Qui-square Test. ^##^Qui-square Test of *worried* (I'm worry a lot, I'm extremely worried) vs. *no worried* (I don't worry, I'm worry a little, I'm not worried). ^a^ Scale from completely unsatisfied (1) to completely satisfied (5). ^b^ Scale from I don't worry at all (1) to I’m extremely worried (5)
