# Supplementary material for: Climacteric women’s perspectives on menopause and hormone therapy: Knowledge gaps, fears, and the role of healthcare advice
Source: PLoS One. 2025 May 9;20(5):e0316873. doi: 10.1371/journal.pone.0316873 (PMC12063881; doi:10.1371/journal.pone.0316873)
Supplement: S2 Table — (DOCX) [file pone.0316873.s004.docx]

**S2 Table.** Attitude of women, according to menopause mode of assessment.

| Characteristics | Total (n=1139) | Post-menopausal women  (n=631, 55.40) | Pre-menopausal women  (n=401, 35.21) | *p*-value |
| --- | --- | --- | --- | --- |
| Age (years), median (IQR) | 51 (12.00) | 55 ( 9.00) | 45 ( 8.00) | <0.001* |
| Menopause, n (%) | 631 (61.14) | 631 (100.00) | 0 ( 0.00) | <0.001# |
| Would you take hormonal medications (MHT) to treat menopausal symptoms? | | | | |
| I wouldn't take it anyway, n (%) | 110 (10.66) | 83 (13.15) | 27 ( 6.73) | <0.001# |
| I don't think I would take it, n (%) | 111 (10.76) | 78 (12.36) | 33 ( 8.23) | <0.001# |
| I don't know if I would take it or not, n (%) | 172 (16.67) | 80 (12.68) | 92 (22.94) | <0.001# |
| I think I would take, n (%) | 295 (28.59) | 150 (23.77) | 145 (36.16) | <0.001# |
| I'm sure I would take, n (%) | 344 (33.33) | 240 (38.03) | 104 (25.94) | <0.001# |
| Why wouldn't you take MHT to treat menopausal symptoms? (conditional answer, n=432) | | | | |
| Afraid of side effects, n (%) | 246 (62.60) | 157 (65.15) | 89 (58.55) | <0.001# |
| They are very expensive, n (%) | 11 ( 2.80) | 10 ( 4.15) | 1 ( 0.66) | <0.001# |
| My partner would not approve, n (%) | 3 ( 0.76) | 2 ( 0.83) | 1 ( 0.66) | <0.001# |
| Don´t believe in the effectiveness, n (%) | 16 ( 4.07) | 13 ( 5.39) | 3 ( 1.97) | <0.001# |
| My gynecologist is against to take MHT, n (%) | 41 (10.43) | 32 (13.28) | 9 ( 5.92) | <0.001# |
| I don't know, n (%) | 76 (19.34) | 27 (11.20) | 49 (32.24) | <0.001# |
| Have you made an appointment with a gynecologist for guidance on menopause or to find out about treatment alternatives in the last 3 years? (n=1139), n (%) | | | | |
| Yes | 522 (50.58) | 396 (62.76) | 126 (31.42) | <0.001# |
| No | 496 (48.06) | 226 (35.82) | 270 (67.33) | <0.001# |
| I don't know | 14 ( 1.36) | 9 ( 1.43) | 5 ( 1.25) | <0.001# |
| At this time, did the gynecologist recommend to initiate MHT? (conditional, n=552), n (%) | | | | |
| Yes | 319 (61.11) | 277 (69.95) | 42 (33.33) | <0.001# |
| No | 199 (38.12) | 117 (29.55) | 82 (65.08) | <0.001# |
| I don't know | 4 ( 0.77) | 2 ( 0.51) | 2 ( 1.59) | <0.001# |
| Which treatments for menopausal symptoms did your gynecologist discuss with you? (conditional, n=325), n (%) | | | | |
| Physical activities, n (%) | 254 (79.62) | 224 (80.87) | 30 (71.43) | 0.226# |
| Yoga, n (%) | 129 (40.44) | 114 (41.16) | 15 (35.71) | 0.616# |
| Acupuncture, n (%) | 95 (29.78) | 82 (29.60) | 13 (30.95) | 1.000# |
| Herbal medicines, n (%) | 161 (50.47) | 140 (50.54) | 21 (50.00) | 1.000# |
| Vitamins, n (%) | 135 (42.32) | 117 (42.24) | 18 (42.86) | 1.000# |
| Hormone therapy (hormone treatment), n (%) | 293 (91.85) | 258 (93.14) | 35 (83.33) | 0.063# |
| None, n (%) | 2 ( 0.63) | 1 ( 0.36) | 1 ( 2.38) | 0.620# |
| I do not know (%) | 2 ( 0.63) | 1 ( 0.36) | 1 ( 2.38) | 0.620# |
| What reasons or indications mentioned below did you initiate taking MHT? (conditional answer, n=325), n (%) | | | | |
| Relieve symptoms such as hot flashes and sweating | 230 (72.10) | 205 (74.01) | 25 (59.52) | 0.077# |
| Relieve sexual symptoms such as loss of libido, vaginal dryness | 173 (54.23) | 151 (54.51) | 22 (52.38) | 0.927# |
| Alleviate emotional symptoms such as anxiety and depression | 147 (46.08) | 137 (49.46) | 10 (23.81) | 0.003# |
| Prevent osteoporosis | 118 (36.99) | 106 (38.27) | 12 (28.57) | 0.298# |
| Prevent cardiovascular disease | 89 (27.90) | 81 (29.24) | 8 (19.05) | 0.235# |
| Prevent dementia | 64 (20.06) | 59 (21.30) | 5 (11.90) | 0.226# |
| To improve the quality of life | 191 (59.87) | 170 (61.37) | 21 (50.00) | 0.218# |
| None of the above reasons would make me use hormones | 19 ( 5.96) | 16 ( 5.78) | 3 ( 7.14) | 1.000# |

*Kruskal-Wallis Test. #Qui-square Test.
