# Supplementary material for: Climacteric women’s perspectives on menopause and hormone therapy: Knowledge gaps, fears, and the role of healthcare advice
Source: PLoS One. 2025 May 9;20(5):e0316873. doi: 10.1371/journal.pone.0316873 (PMC12063881; doi:10.1371/journal.pone.0316873)
Supplement: S3 Table — (DOCX) [file pone.0316873.s005.docx]

**S3 Table.** Practices of women in post-menopausal women (n=631).

| Characteristics | Total (n=631) |
| --- | --- |
| Mean age of menopause (years), median (IQR) | 54 (9) |
| Your menopause (last menstrual period) was? | |
| Spontaneously, n(%) | 542 (85.90%) |
| Oophorectomy, n(%) | 14 ( 2.22%) |
| Hysterectomy, n(%) | 49 ( 7.77%) |
| I don't know, n(%) | 16 ( 2.54%) |
| I can´t remember, n(%) | 9 ( 1.43%) |
| Oophorectomy and Hysterectomy, n(%) | 1 ( 0.16%) |
| At what severity/intensity do you have hot flashes? | |
| Never (not once), n(%) | 79 (12.52%) |
| Rarely (occasionally), n(%) | 145 (22.98%) |
| Neither rarely nor often, n(%) | 60 ( 9.51%) |
| Often (very often), n(%) | 202 (32.01%) |
| Always (every time), n(%) | 145 (22.98%) |
| Have you ever experienced sleep disturbances due to hot flashes? | |
| Never (not once), n(%) | 51 (15.50%) |
| Rarely (occasionally), n(%) | 37 (11.25%) |
| HyNeither rarely nor often, n(%) | 20 ( 6.08%) |
| Often (very often), n(%) | 100 (30.40%) |
| Always (every time), n(%) | 121 (36.78%) |
| Do you have vaginal dryness? | |
| I have, n(%) | 432 (68.46%) |
| I don't have it, n(%) | 162 (25.67%) |
| I don't know, n(%) | 37 ( 5.86%) |
| Do you have pain during sexual intercourse? | |
| I have, n(%) | 307 (48.65%) |
| I don't have it, n(%) | 264 (41.84%) |
| I don't know, n(%) | 60 ( 9.51%) |
| Have you ever lost urine during any type of physical activity or when coughing or sneezing? | |
| I have, n(%) | 324 (51.35%) |
| I don't have it, n(%) | 299 (47.39%) |
| I don't know, n(%) | 8 ( 1.27%) |
| Do you feel a symptom like a burning sensation in vagina? | |
| I have, n(%) | 252 (39.94%) |
| I don't have it, n(%) | 369 (58.48%) |
| I don't know, n(%) | 10 ( 1.58%) |
| Do you have a sleep disturb due to urinary urgency? | |
| I have, n(%) | 413 (65.45%) |
| I don't have it, n(%) | 218 (34.55%) |
| I don't know, n(%) | 0 ( 0.00%) |
| Have you ever taken or are currently taking MHT to treat menopausal symptoms? | |
| Yes, I´ve currently taken, n(%) | 185 (29.32%) |
| Yes, I took hormones in the past and I don't take it anymore, n(%) | 97 (15.37%) |
| No, I never took, n(%) | 345 (54.68%) |
| I don't know, n(%) | 4 ( 0.63%) |
| How long have you been taking hormonal treatment to relieve menopausal symptoms? (n=185, conditional answer) | |
| Less than 1 year, n(%) | 45 (27.95%) |
| Between 1 and 5 Years, n(%) | 94 (58.39%) |
| Between 5 and 10 years, n(%) | 20 (12.42%) |
| I can´t remember, n(%) | 2 ( 1.24%) |
| Which of the following ways of using hormones do you find most comfortable, if you can choose? (Conditional answer, n=282) | |
| Per os, n(%) | 73 (25.89%) |
| Vaginal route, n(%) | 23 ( 8.16%) |
| Percutaneous (gel), n(%) | 64 (22.70%) |
| Transdermic (patch), n(%) | 63 (22.34%) |
| Anyway (indifferent to me), n(%) | 58 (20.57%) |
| I don't know, n(%) | 1 ( 0.35%) |
| What reason (s) did they stop using hormones to treat menopause? (n=282) | |
| Side effects (headache, stomach pain), n(%) | 33 (11.70%) |
| Fear of cancer, n(%) | 10 ( 3.55%) |
| Little improvement in symptoms, n(%) | 10 ( 3.55%) |
| Medication price, n(%) | 2 ( 0.71%) |
| On my own, n(%) | 7 ( 2.48%) |
| By doctor's decision, n(%) | 45 (15.96%) |
| Due to the influence of friends, n(%) | 1 ( 0.35%) |
| Due to the influence of the internet, TV, and newspapers, n(%) | 0 ( 0.00%) |
| I did not stop using hormones to treat menopause, n(%) | 167 (59.22%) |
| I don't know, n(%) | 9 ( 3.19%) |
