# Supplementary material for: Climacteric women’s perspectives on menopause and hormone therapy: Knowledge gaps, fears, and the role of healthcare advice
Source: PLoS One. 2025 May 9;20(5):e0316873. doi: 10.1371/journal.pone.0316873 (PMC12063881; doi:10.1371/journal.pone.0316873)
Supplement: S4 Table — (DOCX) [file pone.0316873.s006.docx]

**S4 Table.** Survey with questions in English version.

|  | Number of responses | Question type | Question | Answer options |
| --- | --- | --- | --- | --- |
| **Seção: Identification** | | | | |
| 1 | 1137 | mandatory question | City |  |
| 2 | 1139 | mandatory question | Age  Date of birth |  |
| 3 | 1137 | mandatory question | Ethnicity | White  Brown  Black  Yellow  Indigenous |
| 4 | 1139 | mandatory question | Marital status | Married  Single  Divorced  Cohabitation/consensual marriage/common-law marriage |
| 5 | 1139 | mandatory question | Do you have children? | Yes  No  If you answered “Yes”, go to question 6  If you answered “No”, skip to question 8 |
| 6 | 1139 | conditional question | Number of children |  |
| 7 | 954 | conditional question | Age of the first childbirth |  |
| 8 | 1139 | mandatory question | Educational degree | Elementary school  Incomplete elementary school  High school  Incomplete high school  Graduation  Incomplete graduation  Post-graduation  Not specified |
| 9 | 1135 | mandatory question | Occupation | Formally employed  Informally employed  Unemployed  Retiree |
| 10 | 1137 | mandatory question | A senhora (ou outra pessoa residente no seu domicílio) recebe algum benefício do governo (ex: BPC, Bolsa Família, Auxílio Brasil etc.)? | Yes  No  I don't know |
| **Seção: Saúde da mulher** | | | | |
| 11 | 1139 | mandatory question | Health insurance | Public  Private  Private and public  None of them |
| 12 | 1139 | mandatory question | Comorbidities self-reported - Do you have? | Hypertension  Diabetes  Cardiovascular diseases  Osteoporosis  Thrombosis  Stroke  Cancer  I don't knowCâncer |
| 13 | 1139 | mandatory question | Gynecological Care (last visit) | Less than 1 year  Between 1 to 3 years  Between 3 to 5 years  More than 5 years  More than 10 years  Never |
| **Seção: Conhecimento sobre a menopausa** | | | | |
| 14 | 1139 | mandatory question | Have you ever heard about menopause? | Yes  No  I don't remember  If you answered “Yes”, go to question 15  If you answered “No” or “I don't remember”, skip to question 27 |
| 15 | 1133 | conditional question | Do you know about menopause symptoms? | Yes  No  I don't remember  If you answered “Yes”, go to question 16  If you answered “No” or “I don't remember”, skip to question 77 |
| 16 | 1068 | conditional question | What symptoms related to menopause are you aware of? | Hot flashes  Vaginal dryness  Mood swings  Sleeplessness  Menstrual irregularities  Loss of libido  I don´t know  **You can select more than one option* |
| 17 | 1139 | conditional question | How did you find out about menopause symptoms? | Physician  Relatives or friends  Internet  Television  Magazines  Newspaper  Teachers  Nurse  Religious leaders  I do not remeber  **You can select more than one option* |
| 18 | 1133 | conditional question | How satisfied were you with the information on menopause that your physician had given? | Completely or reasonably satisfied  I am reasonably satisfied  Neither dissatisfied nor satisfied  I'm a little dissatisfied  I am totally dissatisfied |
| **Seção: Riscos de doenças relacionadas à menopausa** | | | | |
| 19 | 1133 | conditional question | Do you know about risk of having an illness related to menopause**?** | Yes  No  I don't know  If you answered “Yes”, go to question 20  If you answered “No” or “I don't know”, go to question 27 |
| 20 | 654 | conditional question | Which of the follow do you know? | Cardiovascular diseases  Osteoporosis  Dementia  Urinary infection  I don't knowOutro:_________________  **You can select more than one option* |
| 21 | 653 | conditional question | How concerned about the risk of osteoporosis ? | I don´t worry  I'm worry a little  I'm not worried  I'm worry a lot  I'm extremely worried |
| 22 | 654 | conditional question | How concerned about the risk of cardiovascular disease ? | I don´t worry  I'm worry a little  I'm not worried  I'm worry a lot  I'm extremely worried |
| 23 | 653 | conditional question | How concerned about the risk of vaginal dryness ? | I don´t worry  I'm worry a little  I'm not worried  I'm worry a lot  I'm extremely worried |
| 24 | 653 | conditional question | How concerned about the risk of loss of libido ? | I don´t worry  I'm worry a little  I'm not worried  I'm worry a lot  I'm extremely worried |
| 25 | 654 | conditional question | How concerned about the risk of mood swing? | I don´t worry  I'm worry a little  I'm not worried  I'm worry a lot  I'm extremely worried |
| 26 | 653 | conditional question | How concerned about the risk of breast ? | I don´t worry  I'm worry a little  I'm not worried  I'm worry a lot  I'm extremely worried |
| **Seção: Hormônios e tratamento hormonal** | | | | |
| 27 | 1139 | mandatory question | Which of the following do you think women’s bodies naturally produce? | Testosterone  Estrogens  Progesterone  I don´t know |
| 28 | 1139 | mandatory question | To the best of your knowledge, what do you think that happens with hormones after menopause? | Decrease after menopause  Increase after menopause  Doesn’t affect production after menopause  I don´t know |
| 29 | 1139 | mandatory question | Do you know if there is a treatment for menopause symptoms? | Yes  No  I don't know  If you answered “Yes”, go to question 30  If you answered “No” or “I don't know”, go to question 31 |
| 30 | 997 | conditional question | What treatment(s) for menopause symptoms do you know? | Physical activities  Yoga  Acupuncture  Herbal treatments  Vitamin complexes  Hormone therapy  No treatment  I don´t know  **You can select more than one option* |
| 31 | 1138 | mandatory question | What do you know about Menopause Hormone Therapy (MHT)? | I know a lot  I know a little  I don't know anything |
| 32 | 1139 | mandatory question | Do you know if there are negative effects of using hormonal therapy during menopause**?** | Yes  No  I don´t know |
| 33 | 1139 | mandatory question | Do you know if hormonal therapy to treat menopause is offered by the Brazilian health system (SUS)? | Yes  No  I don´t know |
| 34 | 1139 | mandatory question | Do you know if and where the Brazilian Health System offers hormonal therapy to treat menopause? | It is offered at a discount by private health secure  Not offered anywhere  I don't know  In the private system  In the public system (SUS)  In public and private systems |
| **Seção: Atitudes em relação à menopausa** | | | | |
| 35 | 1139 | mandatory question | Would you take hormonal medications (MHT) to treat menopausal symptoms? | I wouldn't take it anyway  I don't think I would take it  I don't know if I would take it or not  I think I would take  I'm sure I would take  *If you answered “I wouldn’t use it at all”, “I don’t think I would use it” or “I don’t know whether I would use it or not”, go to question 36.*  *If you answered “I think I would use it” or “I’m sure I would use it”, skip to question 37* |
| 36 | 432 | conditional question | Why wouldn't you take MHT to treat menopausal symptoms? | Afraid of side effects  They are very expensive  My partner would not approve  Don´t believe in the effectiveness  My gynecologist is against to take MHT  I don't know |
| 37 | 1139 | mandatory question | Have you made an appointment with a gynecologist for guidance on menopause or to find out about treatment alternatives in the last 3 years? | Yes  No  I don't know  *If you answered “Yes”, go to question 38*  *If you answered “No” or “I don't know”, skip to question 41* |
| 38 | 552 | conditional question | At this time, did the gynecologist recommend to initiate MHT? | Yes  No  I don't know |
| 39 | 325 | conditional question | Which treatments for menopausal symptoms did your gynecologist discuss with you? | Yoga  Acupuncture  Herbal medicines  Vitamins  Hormone therapy (hormone treatment)  None  I do not know  **You can select more than one option* |
| 40 | 325 | conditional question | What reasons or indications mentioned below did you initiate taking MHT? | Relieve symptoms such as hot flashes and sweating  Relieve sexual symptoms such as loss of libido, vaginal dryness  Alleviate emotional symptoms such as anxiety and depression  Prevent osteoporosis  Prevent cardiovascular disease  Prevent dementia  To improve the quality of life  None of the above reasons would make me use hormones  **You can select more than one option* |
| **Seção: Práticas em climatério** | | | | |
| 41 | 1139 | mandatory question | Are you in menopause? | Yes  No  I don't know  *If you answered “Yes”, go to question 42*  *If you answered “No” or “I don’t know”, skip to question 55* |
| 42 | 631 | conditional question | Your menopause (last menstrual period) was? | Spontaneously  Oophorectomy  Hysterectomy  I don't know  I can´t remember  Oophorectomy and Hysterectomy |
| 43 | 631 | conditional question | At what age did you go through menopause? |  |
| 44 | 631 | conditional question | At what severity/intensity do you have hot flashes? | Never (not once)  Rarely (occasionally)  Neither rarely nor often  Often (very often)  Always (every time) |
| 45 | 329 | conditional question | Have you ever experienced sleep disturbances due to hot flashes? | Never (not once)  Rarely (occasionally)  Neither rarely nor often  Often (very often)  Always (every time) |
| 46 | 631 | conditional question | Do you have vaginal dryness? | I have  I don't have  I don't know how to inform |
| 47 | 631 | conditional question | Do you have pain during sexual intercourse? | I have  I don't have  I don't know how to inform |
| 48 | 631 | conditional question | Have you ever lost urine during any type of physical activity or when coughing or sneezing? | I have  I don't have  I don't know how to inform |
| 49 | 631 | conditional question | Do you feel a symptom like a burning sensation in vagina? | I have  I don't have  I don't know how to inform |
| 50 | 631 | conditional question | Do you have a sleep disturb due to urinary urgency? | I have  I don't have  I don't know how to inform |
| 51 | 631 | conditional question | Have you ever taken or are currently taking MHT to treat menopausal symptoms? | Yes, I´ve currently taken  Yes, I took hormones in the past and I don't take it anymore  No, I never took  I don't know  *If you answered “Yes, I currently do”, go to question 52*  *If you answered “Yes, I have done it in the past and no longer do it”, skip to question 53*  *If you answered “No, I have never done it” or “I do not know”, skip to question 55* |
| 52 | 185 | conditional question | How long have you been taking hormonal treatment to relieve menopausal symptoms? | Less than 1 year  Between 1 and 5 years  More than 5 years  Between 5 and 10 years  More than 10 years  I don't know |
| 53 | 282 | conditional question | Which of the following ways of using hormones do you find most comfortable, if you can choose? | Per os  Vaginal route  Percutaneous (gel)  Transdermic (patch)  Anyway (indifferent to me)  I don't know |
| 54 | 282 | conditional question | What reason (s) did they stop using hormones to treat menopause? | Side effects (headache, stomach pain)  Fear of cancer  Little improvement in symptoms  Medication price  On my own  By doctor's decision  Due to the influence of friends  Due to the influence of the internet, TV, and newspapers  I did not stop using hormones to treat menopause  I don't know  **You can select more than one option* |
| 55 | 1139 | mandatory question | Which sentence below best represents your opinion about menopause? | All women over 40 will go through menopause at some point and that's okay  I'm afraid of going through menopause  It's a difficult time for women  It's a time to discuss illnesses that can arise at this stage of life  I don't have an opinion about menopause  I wish I had been more informed about this stage of life  None of the above options represent me  **You can select more than one option* |
| 56 | 1139 | mandatory question | How do you rate your knowledge about menopause? | Very good  Good  Average  Bad  I don't know what it is |
| 57 | 1139 | mandatory question | How do you rate your knowledge about the use of hormones to treat menopause? | Very good  Good  Average  Bad  I don't know what it is |
