# Supplementary material for: Climacteric women’s perspectives on menopause and hormone therapy: Knowledge gaps, fears, and the role of healthcare advice
Source: PLoS One. 2025 May 9;20(5):e0316873. doi: 10.1371/journal.pone.0316873 (PMC12063881; doi:10.1371/journal.pone.0316873)
Supplement: Attachment — Submitted filename: Response to Reviewers.docx [file pone.0316873.s008.docx]

**Academic Editor**

Thank you for submitting your manuscript to PLOS ONE. After careful consideration, we feel that it has merit but does not fully meet PLOS ONE’s publication criteria as it currently stands. Therefore, we invite you to submit a revised version of the manuscript that addresses the points raised during the review process.

Congratulations on the study. The work has the potential to advance knowledge about the epidemiology of climacteric syndrome among Brazilian women. Overall, the reviewers' comments were positive, with suggestions for minor revisions throughout the text.

**MAHR: Dear Editor, thank you for your email regarding our manuscript titled “Climacteric *Women’s Perspectives on Menopause and Hormone Therapy: Knowledge Gaps, Fears, and the Role of Healthcare Advice*.” (ID: PONE-D-24-57399). We appreciate the time and effort that you and the reviewers have dedicated to assessing our work. We are grateful for the constructive feedback provided, which will help us improve the quality and clarity of our manuscript. We worked on addressing the points raised during the review process and will submit a thoroughly revised version. If there are any additional suggestions or specific guidelines we should follow during the revision process, please do not hesitate to let us know. Thank you once again for considering our work for publication in PLOS ONE.**

Best regards,

Marcio Alexandre Hipolito Rodrigus

As an academic editor, I believe it would be very interesting if the authors could stratify the analyses not only by menopausal status, but also by the type of health system use, i.e., public vs. private. Approximately 50% of the sample is composed of users of the public system and 50% of users of the private system. It would be important, for example, to know the prevalence of MHT use among users of the Brazilian public system and compare it with the private system.

**MAHR: Thank you for your insightful suggestion. Stratifying the analysis by both menopausal status and type of health system- public and private, is indeed an excellent idea and could provide valuable insights, particularly given the equal distribution of the sample between the two systems. We conducted a preliminary analysis and found a statistically significant difference between the two health systems, which was included in the text (final of results section). However, we think that this is a major issue that should be further explored. Indeed, in addition to the requested information responded to in the manuscript, we are planning to include more data and perform a deeper analysis in the near future. Thank you.**

In addition, to know the opinions about MHT comparing these two groups of women. It was not clear to this reader the difference between “Private w/ Healthcare” and “Private w/o Healthcare”.

**MAHR: Thank you for your feedback. Although we stratified the private system into two subgroups since one of them had a very small number of women, we combined both into a single group as the private healthcare system without compromising statistical analysis.**

1. Please ensure that your manuscript meets PLOS ONE's style requirements, including those for file naming. The PLOS ONE style templates can be found at 
https://journals.plos.org/plosone/s/file?id=wjVg/PLOSOne_formatting_sample_main_body.pdf and 
<https://journals.plos.org/plosone/s/file?id=ba62/PLOSOne_formatting_sample_title_authors_affiliations.pdf>

**MAHR:** **The manuscript has been updated, and the final version now adheres to the style requirements of PLOS ONE.**

2. We note that the grant information you provided in the ‘Funding Information’ and ‘Financial Disclosure’ sections do not match. When you resubmit, please ensure that you provide the correct grant numbers for the awards you received for your study in the ‘Funding Information’ section.

**MAHR: Thank you for bringing this discrepancy to our attention. We have reviewed the details and identified the correct grant numbers associated with the awards we received for this study. When we resubmit the manuscript, we will ensure that the grant numbers are accurately reflected in both “Funding information” and “Financial disclosure” sections for consistency.**

3. Thank you for stating the following in the Competing Interests section. Please confirm that this does not alter your adherence to all PLOS ONE policies on sharing data and materials, by including the following statement: ""This does not alter our adherence to  PLOS ONE policies on sharing data and materials.” (as detailed online in our guide for authors <http://journals.plos.org/plosone/s/competing-interests>).  If there are restrictions on sharing of data and/or materials, please state these. Please note that we cannot proceed with consideration of your article until this information has been declared.

**MAHR:** **The suggested statement was quickly added to the revised manuscript, with track changes highlighted in yellow.**

4. Please include a separate caption for each figure in your manuscript.

**MAHR: We created a separate caption for all the figures from the main manuscript and those were uploaded into the Preflight Analysis and Conversion Engine (PACE) digital diagnostic tool,** [**https://pacev2.apexcovantage.com/**](https://track.editorialmanager.com/CL0/https:%2F%2Fpacev2.apexcovantage.com%2F/1/010f01944f44b418-713d014b-b2de-4ade-8d18-0f2939d3f13b-000000/R15dRLe0ENVMKB8Dg6gO-0sed9CWmKj3eL0PaRrwGUM=192)**. In the supplementary material, the figure captions were updated.**

5. We note that Figure S1 in your submission contain [map/satellite] images which may be copyrighted

**MAHR: We use GeoPandas to illustrate the location provided by volunteers who answered our survey. GeoPandas is an open source library, so we can use it freely without having to ask for permission, as stated in its official documentation available at https://geopandas.org/en/stable/about.html.**

6. Please review your reference list to ensure that it is complete and correct. If you have cited papers that have been retracted, please include the rationale for doing so in the manuscript text, or remove these references and replace them with relevant current references. Any changes to the reference list should be mentioned in the rebuttal letter that accompanies your revised manuscript. If you need to cite a retracted article, indicate the article’s retracted status in the References list and also include a citation and full reference for the retraction notice.

**MAHR: We have included two new references in the current manuscript, numbered 10 and 11 respectively. We have thoroughly checked and updated our entire reference list accordingly (highlighted in yellow).**

**Reviewer #1:** Congratulations to the authors for the excellent research. Women's experience of menopause varies hugely and there is no one-size-fits-all approach to management. Many women transition this stage of life uneventfully, whereas some experience prolonged or severe symptoms and need information, support, or medical treatment. Historically, women have been poorly served by both the research community and by society. It is time for a sensible conversation about menopause to enable informed, individualised decision making on optimal management of this transition, and several medical specialties should be part of this conversation. Understanding our population is the first step to combating misinformation, fear, and deficits in comprehending overall health and comorbidities.

**MAHR: Thank you for your thoughtful feedback and for highlighting the complexity and diversity of women’s experiences with menopause. We completely agree that menopause management must be individualized, as each woman’s journey through this transition is unique. Your point about the historical neglect of menopause in research and societal conversations is particularly important. We hope our research contributes to the broader dialogue and helps empower women to make informed decisions about their health. We also echo your call for a multidisciplinary approach to menopause management, as collaboration between specialties is essential to addressing the wide-ranging impacts of this life stage. Thank you for engaging in this important discussion**

**Reviewer#2:** Abstract
- Objective: Menopause Hormone Therapy- define the acronym MHT on first use of the abstract

**MAHR: The definition of the acronym was added to the revised manuscript, with track changes highlighted in yellow.**

Introduction:

The introduction is quite confusing, both the language and the content.
It is not clear how important it is for menopausal/premenopausal women to know their symptoms and what attitudes are recommended and how they are carried out around the world. I suggest rewriting a few paragraphs with this information that is relevant and supports your work.
**MAHR: Thank you for your valuable feedback. We have revised the paragraphs to effectively emphasize the relevance of this information and its alignment with the overall scope of the work. All changes are highlighted in yellow for your review.**

We two clear objectives in our study: (1) to evaluate women’s during the menopausal transition and (2) to examine how these changes influence their decision-making regarding MHT. This second objective was included in your analysis?
**MAHR: Thank you for your question. Yes, the second objective—to examine how these changes influence decision-making regarding MHT—was included in our analysis. We addressed this in the methodology section when describing the KAP tool. The findings related to this objective are thoroughly discussed in the discussion section, particularly at the beginning of each part concerning knowledge, attitudes, and practices.**

“Unlike two earlier studies conducted in Brazil [18,19], before the pandemic period, our survey included premenopausal and postmenopausal patients aged between 40 and 65 years” – Did these Brazilian studies have the same objective as this study? Or did the findings of these previous studies support the decision to carry out this study?
**MAHR: These two Brazilian studies were conducted prior to the pandemic period and did not include premenopausal women. Therefore, our study aimed to evaluate patients after this period, including a broader patient demographic, specifically both premenopausal and postmenopausal women aged 40-65 years, and addressing changes during the pandemic period, which was not a focus in the earlier studies.**

Methods
- I suggest removing the period: “This tool was used in this study” and using the reference in the previous period.
**MAHR: This statement was removed from the new version while the reference was shifted to align with the updated manuscript. All track changes were highlighted in yellow.**

- it is not clear that the answers to the questions are arranged on a Likert scale, nor how the authors used to assess the correlation between the answers (Reading the Results section, in Knowledge, there was a categorization to assess the degree of women’s satisfaction and the level of concerns related to menopausal period)

**MAHR: Thank you for your insightful comment. To clarify:**

**1. Use of the Likert Scale: The answers to the questions in the survey were indeed arranged on a Likert scale. We have now explicitly mentioned this in the Methods section of the manuscript highlighted in yellow. You also can check the scale in supplementary Table 1 as well as in question 18 of the original version of the questionnaire in Portuguese. For example, responses regarding satisfaction and concerns were rated on a 5-point Likert scale (e.g., ranging from Completely satisfied, reasonably satisfied, I am reasonably satisfied, neither dissatisfied nor satisfied, I'm a little dissatisfied). The first two answers were analyzed together.**

**2. Correlation Assessment: In assessing the correlation between responses, we used the Qui-square Test. This has been clarified in the Methods, Results sections, and supplemental material, including details of how the correlation coefficients were interpreted.**

**We hope this additional explanation resolves the concern and have revised the manuscript accordingly to ensure clarity. Thank you again for your feedback.**

- the healthcare providers are only gynecologists or general practitioners?
**MAHR: the healthcare providers include gynecologists and general practitioners, which were predominantly in the public system.**

Results
Table 1:
- organize the table, making it uniform. For example, how to put “n/N (%)” in the columns and not in the rows [Total (n (%))]
- approximate the percentages so that the total is 100%. In “Occupation” for “Premenopausal Women” the sum is 100.01%
- please, describe the statistical test used
**MAHR: 1. Organizing the table: Thank you for your feedback. I will revise the table to ensure uniformity by presenting the “n/N (%)” format consistently in the columns rather than in the rows. The updated table will align with your suggested format for clarity and readability. 2. Approximating percentages: I will ensure the percentages are rounded appropriately so that the total equals 100%. In the case of ‘Occupation’ for ‘Premenopausal Women,’ the rounding issue resulting in 100.01% will be corrected; 3. Describing statistical test: I will include a description of the statistical test used in the analysis. All track changes were highlighted in yellow.**

- In the results section, it is necessary to present the findings in your research. Therefore, I suggest reviewing and removing the adverbs of intensity (surprisingly, notably).

**MAHR: Thank you for your valuable feedback. I understand the importance of maintaining an objective tone in the results section. I will carefully review the text and remove the adverbs of intensity, to ensure the findings are presented in a neutral and precise manner. All changes have been marked and highlighted in yellow.**

**Reviewer #3:** This was a well-designed study, with an expressive number of women assessed, highlighting an important theme, which will affect even more women around the world.

**MAHR: Thank you for your thoughtful feedback. We are glad you found the study well-designed and impactful. Addressing such a significant theme with a large sample of women was a priority for us, as we aimed to provide meaningful insights that could have a global impact.**

I have only minor comments to do:

- Introduction: at the second paragraph fourth line - the utilization of this treatment use (sounds confusing as a sentence)

**MAHR: the second paragraph was re-structured.**

- Methods - also present at the abstract, the dates are not in international Standard. The year is written first, then the month, then the day. This is also known as the YYYY/MM/DD format

**MAHR: Thank you for your observation. I revised them to ensure compliance with this format.**

- At the sample size calculation there is a sentence where "864 women will be required" suggestion - 864 would be required to conduct

**MAHR: the sentence was corrected and highlighted in yellow.**

- Ethical considerations - Considering there are two centers, where are the approval of the second one?

**MAHR: Yes, the study was approved in the Institutional Review Board of both Centers. At Center I, coordinated by the Department of School of Medicine at UFMG, we used the approval protocol from the Ethics Committee of USP in São Paulo. Please, see attached the approval from the Department of Gynecology of the aforementioned School at UFMG, as well as the approval from the Hospital das Clínicas of this institution.**

Results:
At the table 1 it is written: Total %), lacking a parenthesis, and there are spaces between the parenthesis and some of the numbers - only suggestions of details
As it was done in table 2 - suggest in table 1 to put the statistical tests used

**MAHR: Thank you for your observations. Regarding Table 1, We corrected the missing parenthesis and adjust the spacing issues to ensure consistency. Additionally, we also will include the statistical tests used in Table 1, following the format used in Table 2. We appreciate your detailed suggestions.**

No further considerations
The discussion was brilliant, separating and organizing the writing like the way you did, only makes the reading better

**MAHR: Thank you so much for your kind words! I´m glad you enjoyed the discussion and the way it was organized. It´s great to hear that the structure made the reading experience better – your feedback means a lot!**
