# Supplementary material for: Climacteric women’s perspectives on menopause and hormone therapy: Knowledge gaps, fears, and the role of healthcare advice
Source: PLoS One. 2025 May 9;20(5):e0316873. doi: 10.1371/journal.pone.0316873 (PMC12063881; doi:10.1371/journal.pone.0316873)
Supplement: Attachment — Submitted filename: Response to Academic Editor.docx [file pone.0316873.s009.docx]

Response to Academic Editor,

Congratulations on the corrections made to the article. The reviewers' questions were adequately answered and the changes to the manuscript are appropriate. However, I suggest correcting the following points before possible publication.

In the section “We analyzed the relationship between public and private health systems and the use of hormone therapy (HT) for menopause treatment. Our findings indicate that women using the public health system are less likely to use HT (62.59% out of 270) compared to those using the private system (49.78% out of 233), representing a difference of 12.81%. To assess the statistical significance of this observation, we performed a Chi-Square test, which yielded a p-value of 0.0168. Given a significance level of 0.05 (95% confidence), we can reject the null hypothesis and conclude that these results are unlikely to be due to chance. Further research will explore the broader impact of public and private health systems on treatment choices.” I believe there was a typing error, since, from what can be understood from what was written, women in the public system used more HT. I suggest reviewing it.

#1. Author´s response

**Thank you for your thorough review and valuable feedback. We appreciate your attention to detail. We will carefully verify the data and wording to ensure accuracy and clarity. Please take a moment to review the modified text:**

**Our results highlight an important observation regarding women’s usage of hormone therapy (HT) across different healthcare systems. Specifically, 62.59% of women who rely on the public health system (from a sample of 270) are less likely to use HT, compared to 49.78% of those in the private system (from a sample of 233) representing a difference of 12.81%. To assess the statistical significance of this observation, we performed a Chi-Square test, which yielded a p-value of 0.0168. Given a significance level of 0.05 (95% confidence), we can reject the null hypothesis and conclude that these results are unlikely to be due to chance. Further research will explore the broader impact of public and private health systems on treatment choices.**

In Table 1, I suggest making it clear that there is missing data for some women since the sum of the absolute numbers in the rows does not always correspond to the total number evaluated.

#2. Author´s response

**Thank you for your suggestion. We acknowledge that there is missing data for some women, which is why the sum of the absolute numbers in the rows does not always match the total number evaluated. We will clarify this in Table 1 including this information as a footnote to ensure transparency.**

**Footnote: The absolute number of responses for each question does not represent the total sample pf 1,139 participants due to missing data.**
